# Temporal characterization of biocycles of mycelium-bound composites made from bamboo and *Pleurotus ostreatus* for indoor usage

**DOI:** 10.1038/s41598-022-24070-3

**Published:** 2022-11-12

**Authors:** Jun Ken Gan, Eugene Soh, Nazanin Saeidi, Alireza Javadian, Dirk E. Hebel, Hortense Le Ferrand

**Affiliations:** 1 School of Physical and Mathematical Sciences, Nanyang Technological University, 21 Nanyang Link, Singapore, 637371 Singapore; 2 School of Mechanical and Aerospace Engineering, Nanyang Technological University, 50 Nanyang Avenue, Singapore, 639798 Singapore; 3 Faculty of Architecture, Karlsruhe Institute of Technology, Engelstrasse 11, 76131 Karlsruhe, Germany; 4 School of Materials Science and Engineering, Nanyang Technological University, 50 Nanyang Avenue, Singapore, 639798 Singapore; 5 Future Cities Laboratory, Singapore ETH Centre, 1 Create Way, Create Tower #06-01, Singapore, 138602 Singapore

**Keywords:** Materials science, Biomaterials

## Abstract

Mycelium-bound composites (MBCs) are materials obtained by growing fungi on a ligno-cellulosic substrate which have various applications in packaging, furniture, and construction industries. MBCs are particularly interesting as they are sustainable materials that can integrate into a circular economy model. Indeed, they can be subsequently grown, used, degraded, and re-grown. Integrating in a meaningful biocycle for our society therefore demands that MBCs fulfil antagonistic qualities which are to be at the same time durable and biodegradable. In this study, we conduct experiments using MBCs made from the fungus species *Pleurotus ostreatus* grown on bamboo microfibers substrate. By measuring the variations of the mechanical properties with time, we provide an experimental demonstration of a biocycle for such composites for in-door applications. We found that the biocycle can be as short as 5 months and that the use of sustainable coatings is critical to increase the durability of the composites while maintaining biodegradability. Although there are many scenarios of biocycles possible, this study shows a tangible proof-of-concept example and paves the way for optimization of the duration of each phase in the biocycle depending on the intended application and resource availability.

## Introduction

**Figure 1:**
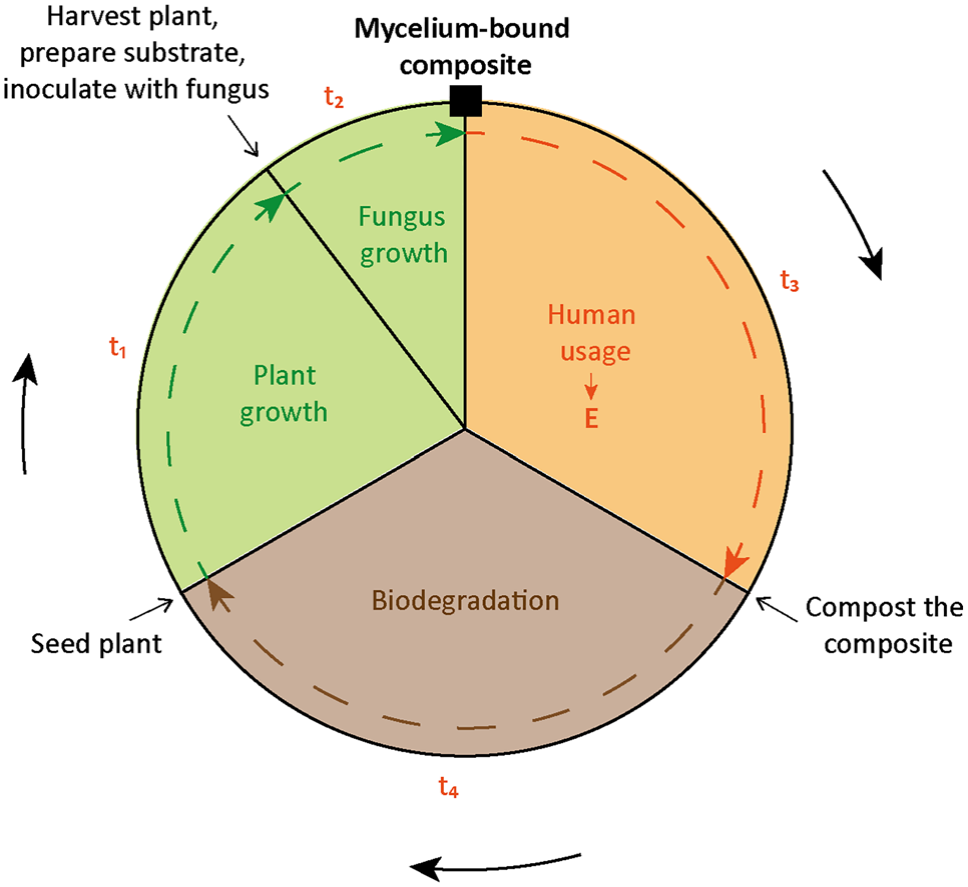
Life cycle of MBCs. *E* represents the elastic modulus and *t*_*i*_ is the time for the phase *i* of the cycle: 1, growth of the plant that will make the ligno-cellulosic substrate, 2, growth of the mycelium, 3, usage, and 4, biodegradation in soil.

Mycelium-bound composites (MBCs) are obtained by growing a fungus onto a solid ligno-cellulosic particulate-based substrate. When growing, the fungus develops a highly branched network of cells, called mycelium, that binds the particles from the substrate together, yielding a composite material. MBCs are attracting attention thanks to their properties, such as resistance to flammability^1^, sound absorption^2^, compression properties^3,4^, and their potential to be part of a circular bioeconomy^5,6^. Indeed, a large diversity of waste-based substrates have been found to be able to grow mycelium on them, such as plants' leaves, diapers, paper, food waste, etc^7–12^, therefore promising the production of new materials without the need for depleting resources. Also, contrary to other sustainable alternatives which are plant-based, MBCs can grow on by-products which may reduce the need for land, and contrary to other sustainable alternatives based on recycling of synthetic materials, MBCs require low energy input and produces low CO_2_ emission which could be further reduced by employing ambient growth conditions for the fungus^13^. MBCs can integrate into biocycles because they are biodegradable^14–16^. Yet, MBCs are aimed for applications such as packaging, insulation, as well as decorative and even architectural elements^17,18^. For these applications, MBCs also need to be durable. Architectural structures made from MBCs and exposed outdoors were shown to retain their integrity for 6 weeks, whereas after 4 months, cracks, decay, infestation, and contamination with potentially harmful organisms were observed^19,20^. A circular bioeconomy incorporating MBCs could include 4 main phases (Fig. 1): (1) the production of the materials required for the substrate onto which the fungus will grow, (2) the growth of the mycelium to form the composite, (3) the usage of the MBC, (4) its biodegradation in soil before being reused to regrow mycelium and produce a new MBC. To demonstrate such circular biocycle and assess its relevance for a circular bioeconomy, there is thus a need to experimentally quantify the duration of each phase of the biocycle.

To date, MBCs are deemed sustainable because they are biodegradable, demand 1.5 to 6 time less energy for their fabrication as compared to other materials, can act as CO_2_-sink, and have reduced impacts on water usage, particle emissions and overall climate change^13,21^. Yet, their integration into a tangible biocycle is still pending. There is little experimental testing reporting on the durability of MBCs. Chan et al. conducted accelerated weathering on compressed MBCs and found a decrease of 82% of their elastic modulus after 35 days^22^. The addition of a commercial, eco-friendly coating reduced the decrease in elastic modulus down to only 52% after 35 days. Similar to the durability, the biodegradation of MBCs in the environment and their reuse is scarcely documented. Van Wylick et al. reported a decrease in mass of the composites by 43% after 16 weeks in soil^14^. Other approaches of recycling and reuse of the composites have been explored, for example for bioremediation, animal feed, and energy feedstock^23,24^, as well as for new cultivation of fungi after supplementation with wheat bran and soy bean flour^25,26^. However, there is no experimental demonstration of the reuse of MBCs for making new composites. There is thus a need to further quantify the durability of MBCs, their biodegradability, and thereby to demonstrate their integration in a circular biocycle.

The aim of this study is therefore to quantify the duration of each of the 4 phases of the biocycle in Fig. 1 and the mechanical properties of MBCs made by growing *Pleurotus ostreatus* on a bamboo-based substrate. *P. ostreatus*, also called the Oyster mushroom, was selected as the fungal species for this study because it is among the most studied and used fungus for making MBCs^12,27,28^. Bamboo was used as the substrate because bamboo is a plant with one of the highest growing rate, about 30–100 cm per day^29–31^. Also, bamboo is a grass that grows in all continents of the planet and that is also harvested for making biofuels, food, or for architecture and construction^32^, and that has been found successful for growing fungi^12^. After describing the experimental methods, each phase of the biocycle is described, and their duration quantified using mechanical testing. Given the limited time available for the work, the durations that will be studied are minimum durations, which are minimum durations for growing the mycelium to produce the composite, for acceptable in-door storage of the MBCs, for degradation in soil, and for growing the bamboo plant to be used as the substrate. First, fresh MBCs are grown. Second, the shelf-life of raw composites as well as for composites coated with beeswax, and a commercial waterproof spray is monitored for 2 months. Third, the composites with and without coating are composted in soil for 2 months. Finally, a used MBC is mixed with new fresh substrate to grow a second cycle of MBCs. The growth of plants using the degraded substrate as compost is not experimentally conducted in this work, but others have done so for a variety of plants, such as tomato, pepper, cucumber plant, etc^33–35^. Overall, with this experimental demonstration of a biocycle based on MBCs and the quantification of the duration of each phase, researchers and developers can conceive MBCs to be part of the biocycle in the most efficient way. Also, it can provide a benchmark for stakeholders and customers interested to invest in MBCs by integrating the biocycle into a circular bioeconomy.

## Materials and methods

### Materials

Spawn from the Oyster mushroom *P. ostreatus* was obtained from a local supplier, Bewilder, Singapore. Bamboo fibers from *Dendrocalamus asper* were obtained from bamboo sheets (Widuz, Singapore) that were dried at 80 °C using a curing oven (Qingdao Gupsen-1800, China), grinded using a cutting mill (Fritsch pulverisette 15) and sieved using a vibratory sieve shaker (Fritsch analysette 3 spartan) to obtain fibers of 200 µm in length on average. To accelerate the initial growth of the mycelium, commercially available quick cook oats (Quaker, 100% Australian wholegrain oats, product of Malaysia), bought from the local supermarket were ground up using a blender mixer (Powerpac). Deionized water was used to provide the moisture and water content to the growing composites. Natural beeswax (Furniture oil, Dicon, Singapore) and commercial waterproof spray (Nano Protector, Eykosi, Malaysia) were used for the coatings. Mesh teabag pouches (Daiso, Japan) and potting soil mix (Hua Hng, Singapore) were purchased to do the composting experiments.

### Mycelium-bound composite preparation (1st cycle)

**Figure 2:**
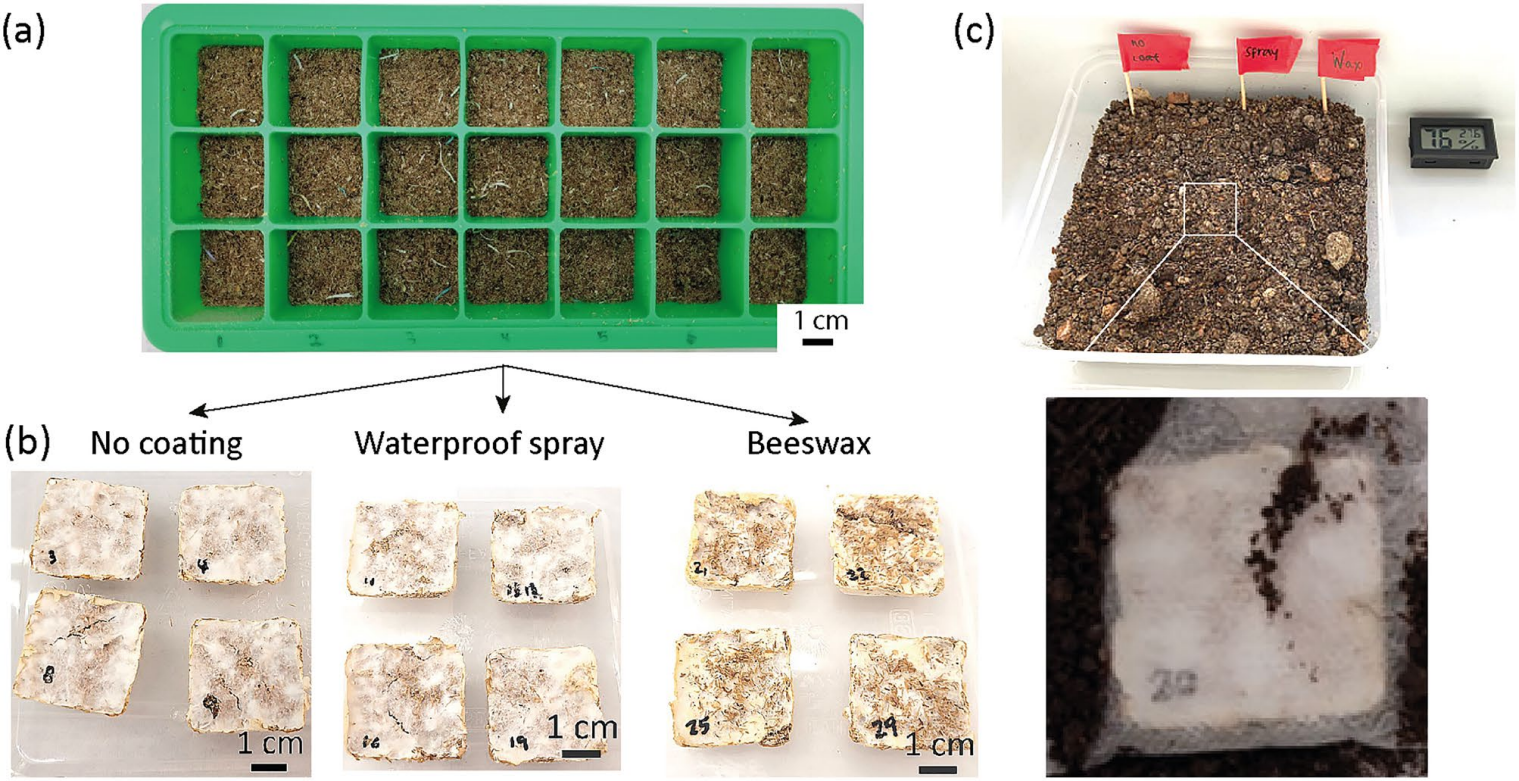
Experimental set-up. (**a**) Picture of the substrate placed in the ice tray molds at day 0 for the growth of the MBCs. (**b**) Pictures of the MBCs after 28 days fungal growth and drying, with no coating, waterproof spray coating and beeswax coating. (**c**) Experimental set-up for the composting showing the soil with outdoor tropical conditions and a picture of a MBC sample placed inside a pouch before its burial in the soil.

For the fabrication of the fresh MBCs, solid-state fermentation was used where the fungus is grown on a solid substrate. This substrate was composed of 30 g of bamboo fibers, 15 g of oats and 45 ml of deionized water. This substrate mixture was mixed thoroughly before being sterilized in an autoclave (Hiclave HG 80, Hirayama, Japan) at 121 °C for an hour. Once cooled, 30 g of the mycelium spawn was added to the organic substrate at a mass ratio spawn: substrate of 1:1 and placed into an ice cube tray and lightly compacted to form a cuboid shape of dimensions 3 × 3 × 1 cm^3^ (width x breadth x height) (Fig. 2a).

The samples were then kept in a cupboard in the dark for 28 days at a room temperature of 22 °C and a relative humidity of 80% with periodic misting with deionized water, for the fungus to develop and grow its mycelium. 28 days was chosen as the maximum time for growth as longer time leads to the development of fruiting bodies instead of the mycelium. Also, it is reported the mycelium network and the mechanical properties improve till 28 days for this substrate composition^12^. A total of 30 samples was fabricated with a contamination rate of only 3%, showing the efficacy of the growth conditions.

### Coating and durability

To improve the durability of the MBCs, a coating was added on samples grown for 28 days after drying in oven (Ika, Germany) for one day at 48 °C. The waterproof spray coating was applied as an aerosolized spray from 30 cm away on all sides of the samples. The beeswax coating was applied on all sides of the sample using a brush. After the application, the samples were left in air for 48 h for the coatings to solidify. Out of the 29 successfully grown samples, 10 samples were coated with the waterproof spray, 10 samples with beeswax, and 9 samples remained uncoated as control samples (Fig. 2b). To assess the durability of the MBCs, 3 samples from each group were placed in a cupboard in the dark and in ambient conditions (room temperature of 22 °C and relative humidity of 80%). The samples were kept in these conditions for 60 days until they were tested under compression.

### Composting

To assess the effect of degradation in soil of the MBCs, the following series of samples were prepared: 3 samples with no coating, 3 samples with beeswax coating and 3 samples with waterproof spray coating and were individually sealed within mesh pouches and buried in a bed of potting soil at an approximative depth of 2 cm (Fig. 2c). The soil bed was left exposed in a naturally ventilated area in the tropical climate of Singapore but in an area protected from natural rain. The experiment was carried out under average temperature and humidity of about 29 °C and 80%. Rainfall was simulated by the addition of 100 ml of tap water every week to the soil bed. The samples were left buried for 60 days before they were removed and dried for a day in an oven at 48 °C.

### Mycelium-bound composite preparation (2nd cycle)

After the MBCs had been kept for in-door usage for 2 months and submitted to the compression test, they were crushed into loose particles. The obtained particles were mixed at a ratio of 1 to 1 with fresh bamboo fibers to make up a new substrate to grow mycelium. The mycelium growth was carried out as described for the composites of the 1st cycle. After the 28 days growth, 6 samples (2 with no coating, 2 with the waterproof spray and 2 with beeswax) were successfully grown and dried in oven and tested in compression without addition of any coating.

### Characterization and testing

Macroscopic images were taken with the camera from a smartphone. Microscopic images were also obtained after breaking the MBCs, drying them overnight at 48 °C, and observing them using a scanning electron microscope (SEM, JSM-5510LV, JEOL, Japan) after 45 s of sputtering with gold using a Cressington 108 Gold Sputter Coater, UK.

The mechanical properties of the samples were investigated in compression using an Instron Universal Testing Machine (Instron 5567, USA) at a strain rate of 3 mm/min till 40% strain was reached. Triplicates were done for each type of composite. The elastic modulus *E* was obtained from the stress–strain curves by taking the gradient at 10% strain for all composites. Since the samples densified during compression, the linear region of the curves was difficult to determine. By fitting the slopes of the stress–strain curves around 10%, a linear fit with a coefficient of correlation around 0.99 could be obtained and reliably used to compare the samples. The compression tests at different growing time of the mycelium were conducted on the samples at the relevant time point, without drying. For the evaluation of the effects of the coatings and for the MBCs obtained after the second cycle, the samples were dried in oven before the testing.

Hydrophobic properties of the samples were observed using a portable microscope (Dino-lite AM7915MZTL). A 10 μL drop of water was placed onto a random surface of the sample and left undisturbed. Pictures of the droplets were recorded for 20 min, and the contact angle of the droplets were measured using Image J. The tests were repeated 3 times.

The mass of the samples was recorded using a conventional scale (ML 304 T, Mettler-Toledo). Prior to measuring the mass, the samples were dried in an oven at 48 °C for 2 days. The samples that became loose and powdery after the degradation could not be measured.

## Results and discussion

### First cycle

#### Phase 1: bamboo growth

Bamboo is a grass growing in most regions of the world, including tropical and subtropical regions as well as temperate climates^36^, and used for a large variety of applications including food, construction, textiles, paper, charcoal, etc^37^. What makes bamboo particularly interesting is its fast-growing rate and high mechanical properties for a low weight. For example, the Moso bamboo, also called giant bamboo and one of the most popular types of bamboos, can attain 10–20 m height and 8–16 cm diameter in only 35–40 days. The growth speed of Moso bamboos is referred to as “explosive speed” which can attain 0.5 m/day^38^. The mechanical properties of bamboo are also known for their high strength and toughness^31^, and bamboo microfibers are regularly used as reinforcement in composites^39,40^. The duration ${t}_{1}$ of this phase of the cycle can be expected to last between 1 and 2 months to grow a bamboo stem that can be processed into fibers for making the substrate to grow mycelium.

To get a more specific estimation of the amount of bamboo and of the time for its growth ${t}_{1}$ for MBCs of sizes like those used in this study, 3 × 3 × 1 cm^3^, simple calculations can be made. Assuming a bamboo stem of 15 m in height, 12 cm of outer diameter, 8 cm of inner diameter and a porosity of 50%^41^, the total volume of ligno-cellulosic material is 47,100 cm^3^ (0.0471 m^3^). Assuming a loss of 20% of the material during the process to transform the bamboo stem into bamboo microfibers due to cutting, steaming, pulverizing, and sieving, the total volume of ligno-cellulosic material decreases to 37,680 cm^3^. With a theoretical density of cellulose of 1.5 g cm^-3^, this volume produces a mass of about 56 kg of bamboo microfibers, which could produce 1884 samples of size similar to those made in this study. This calculation also shows that a bamboo stem of 1.59 cm length, 5 mm outer diameter and 3 mm inner diameter is sufficient to make one sample. With a growing speed of 16 cm/day, a common growing rate for bamboos^42^, attaining 1.59 cm length for a bamboo stem would only take 2.3 h. Such a short time may not be realistic but illustrates well the interest of using bamboo as a substrate for growing MBCs.

In practice, the bamboo material used for growing the MBC can be the waste or byproduct from bamboo industries. Therefore, there is an opportunity to integrate the MBC biocycle with other biocycles based on bamboo. Although the focus in this study is on bamboo, other plants can be used as well. Overall, a realistic time for this first phase can therefore be of about 2 months. Bamboo plants are generally harvested after about 2 years of growth for their strength; therefore, such a short time could be for growing bamboo stems only to serve as nutrients for the fungus. After preparing the bamboo microfibers, the MBC can be grown. The preparation of the bamboo microfibers and their sterilization can be estimated to an additional time of about 1 week with drying of the bamboo taking the longest time in this process.

#### Phase 2: growth of the mycelium-bound composite

**Figure 3:**
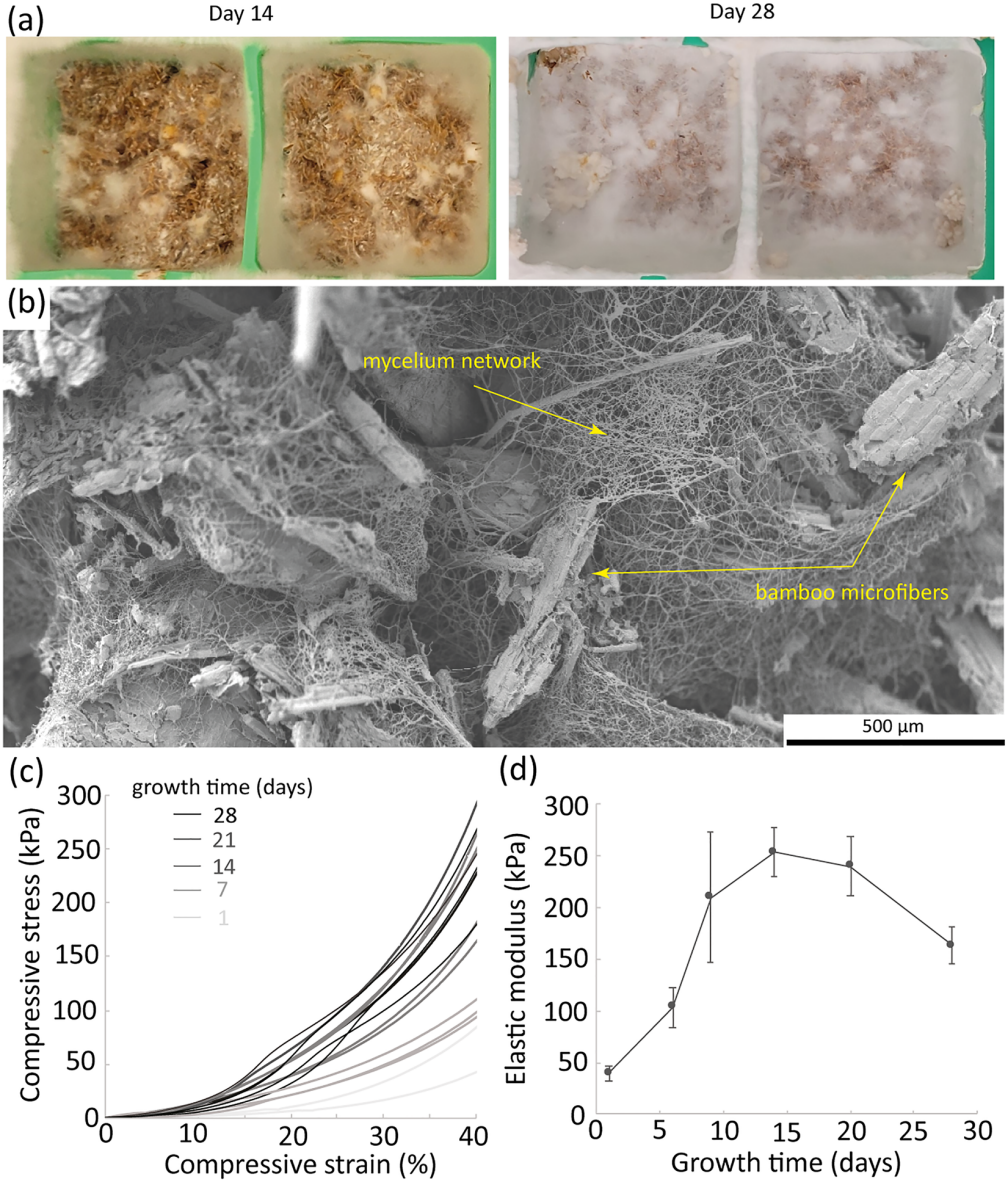
Growth of the mycelium-bound composite. (**a**) Pictures of mycelium-bound composites in their green ice-cube tray mold, after 14 and 28 days of growth. The width of the composites is 3 cm. (**b**) Electron micrograph showing the internal structure of the composite with the mycelium network binding the bamboo microfibers after 1 week of growth. (**c**) Stress–strain curves obtained during compression of the composites at different time points of the growth. (**d**) Elastic modulus as a function of the growing time, measured at 10% of the compressive strain.

After the ligno-cellulosic substrate is prepared using the bamboo microfibers, a MBC can be produced. Here, *P. ostreatus* was chosen as the fungus due to its popularity in MBCs research. The substrate made of the bamboo microfibers was supplemented with oats as a source of sugar and nutrients, moisturized and inoculated with the spawn. Visual inspection and mechanical properties as a function of the growth time of the mycelium were recorded to evaluate the time needed to obtain the composite (Fig. 3).

As *P. ostreatus* grows on the substrate, it develops its mycelium network that binds the particulates of the substrate together and is optically visualized as a white material (Fig. 3a). After 21 days, the composites were entirely covered with mycelium, indicating that the substrate was adequate for the growth of the fungus (Fig. 3b). Indeed, the bamboo microfibers provided the cellulose content that is degraded and digested by the fungus, whereas the oats provided extra nutrients. The growth was continued until 28 days maximum as the fungus started to develop fruiting bodies, the mushrooms, at longer growing times. The growth of fruiting bodies indicated that the mycelium had consumed most of the nutrients and was producing spores to spread to other substrates. For our growth conditions, it therefore required a maximum of 28 days to grow MBCs. This time could be reduced by providing a more nutrient-rich substrate, optimized growth conditions and fungal strain, although most MBCs reported in the literature are also grown between 15 and 30 days^43^.

As MBCs are used for various applications, their mechanical properties are an important characteristic. Compressive tests were therefore conducted for composites at different time points in their growth in order to follow the strengthening of the material with growing time. The stress–strain curves obtained showed a typical densification behavior (Fig. 3c). The curves of the composites grown for 21 and 28 days exhibited a shoulder with a change in curvature around 15–20% strain, suggesting the appearance of a yield stress at about 60–100 kPa. The slope of the curves increased after this yield stress. At low strains, the mycelium network likely held the particles from the substrate together while being compressed, whereas past the yield, the mycelium network likely broke, facilitating the densification of the composite. The compressive stresses increased with the mycelium growth time.

The elastic moduli of the composites were measured at around 10% strain, where the stress–strain curves were linear, and were found to increase from 50 to 250 kPa for composites grown until 14 days (Fig. 3d). At 21 and 28 days, the moduli were found to be lower than for day 15. This is consistent with the stress–strain curves where the compressive stresses of the composites at 40% strain tended to be lower for the composites grown at 21 and 28 days, as compared to those grown till day 14. A reason for the decrease in stiffness after 2 weeks growth could be the increase in water content as more mycelium has developed. Another reason could be the weakening of the particulate substrates as they were getting further degraded. Therefore, the best growth time for a stronger MBC in the conditions of this study is 14 days. Nevertheless, longer timings show more mycelium at the surface of the composite, called fungal skin, as evidenced in the images in Fig. 3a. More fungal skin gives a better surface finish of the composite, depending on the intended application. The compressive modulus measured here are about 4 times lower than other MBCs of similar composition^44,45^ which is likely due to the fact that the MBCs here are not dried before the mechanical test, and about 10 times lower than similar MBCs after cold or hot-pressing^3^.

The time ${t}_{2}$ for the second phase of the MBC biocycle therefore spans between 15 and 28 days. After that time the composite are dried and used in indoor conditions.

#### Phase 3: indoor usage of the composite/shelf life

**Figure 4:**
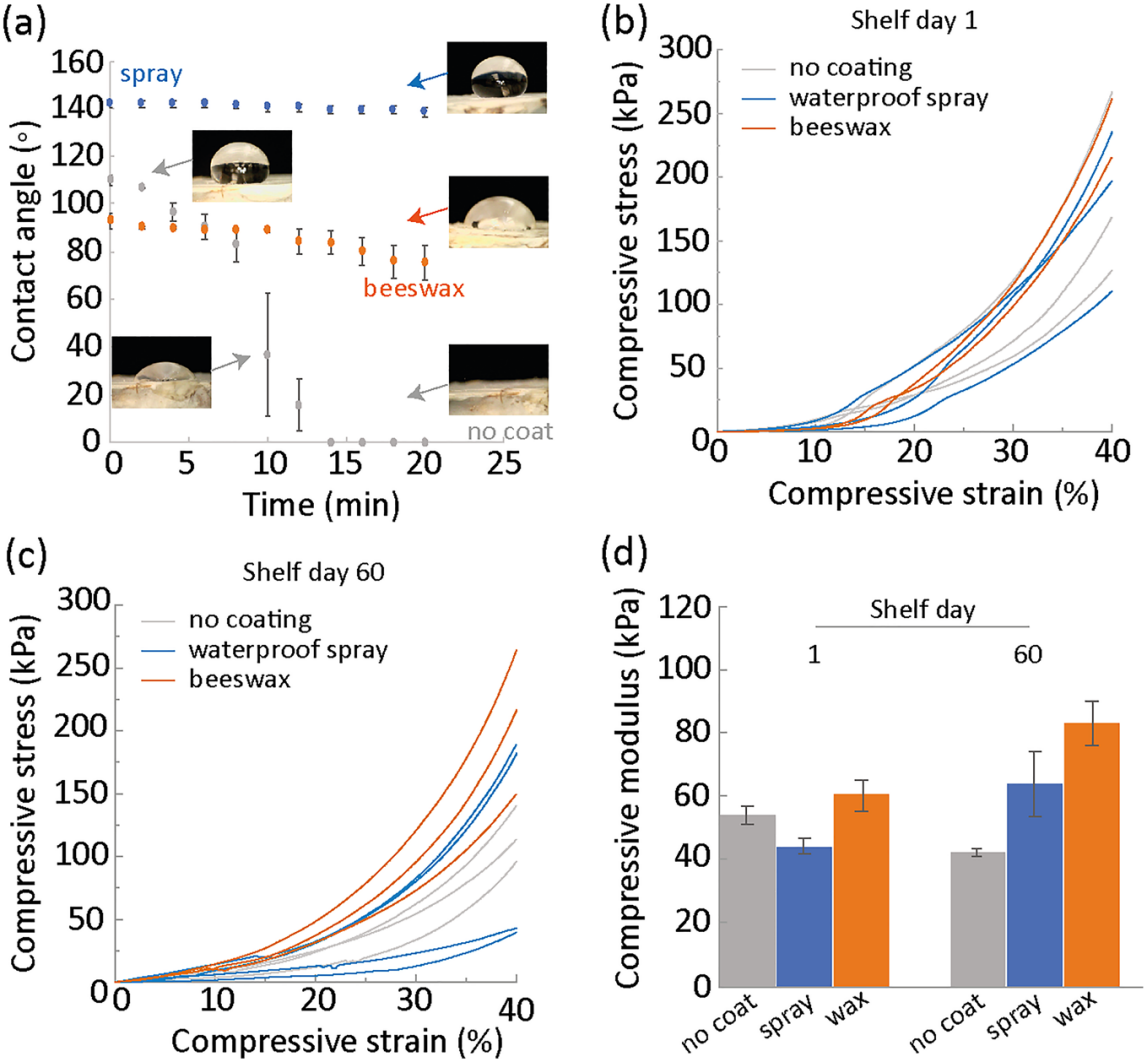
Indoors durability for MBC with no coating (grey), waterproof spray coating (blue) and beeswax coating (orange). (**a**) Contact angle as a function of time after a drop of water was deposited at the surface of the samples. The images show the morphology of the drop at several time points. (**b**) Stress–strain curves obtained under compression for the MBC grown for 28 days, dried, and coated or non-coated. (**c**) Stress–strain curves obtained under compression for the MBC after 60 days indoors shelf storage. (**d**) Elastic modulus of the samples after 1 and 60 days indoors for the different coatings.

To demonstrate that MBCs can be used for a meaningful time, the fabricated composite were first dried to stop the growth of the fungus. In view of their usage indoors, the MBCs need to be protected against insects and humidity so that they maintain their properties. To help increase the durability, a coating was applied at the surface of the composites, on the fungal skin, to make them more hydrophobic. Coating is a common strategy to protect materials and increase their durability^46^. Two types of coatings were used: a natural biodegradable material, beeswax, and a commercial waterproof spray of unknown composition. The coated and uncoated MBCs were then left on a shelf indoors for 60 days and their durability was assessed by compression tests (Fig. 4).

Two hydrophobic coatings were applied onto the surface of the composites to increase their durability indoors. The coatings could be easily applied on the fungal skin at the surface of the composites which the fungus grows to help retain the moisture^47^. Although the fungal skin has slightly hydrophobic properties, with a contact angle with water of about 110$^\circ$, it is still able to absorb water. It was found that a drop of water deposited onto the fungal skin was absorbed in less than 15 min (Fig. 4a). With the beeswax coating, the surface remained slightly hydrophobic with a slow decrease in contact angle from 90 to 80$^\circ$ in about 20 min. The waterproof spray was the most hydrophobic, with a contact angle of 140$^\circ$ and no observable absorption after 20 min. The high performance from the commercial spray is expected as it contained nano waterproof and antifouling agents and solvents, whereas beeswax is a natural compound that contains a small amount of water of 0.5%. However, beeswax is more sustainable than the commercial spray and also possess natural antimicrobial activity^48^.

Right after drying and applying the coatings, there was no significant difference in the mechanical properties of the MBCs (Fig. 4b). The stress–strain curves exhibited the same compression behavior as discussed previously. All samples exhibited a yield point between 15 and 25% strain for stress values between 25 and 50 kPa. These stress values are lower than for the samples before drying and must result from the water loss that made the mycelium more brittle and fragile. The absence of effects from the coating is likely due to its small thickness and weak properties. Indeed, the coating materials had been chosen for their hydrophobic properties and not for their mechanical properties.

After leaving the composites for two months on a shelf indoors, the samples retained the same physical appearance as before and no infestation with insects was visible. The compressive properties were measured and compared to the samples before the two months exposure (Fig. 4c). The compression curves again showed the densification behavior but did not display the curvature change between 15 and 25%. Although the compressive stresses at 40% strain were slightly lower as previously measured, between 0 and 10% strain, the compressive stresses were superior, especially for the samples coated with beeswax. These results therefore suggest that after two months on a shelf, there might have been some microstructural changes within the composites. Redoing the hydrophobicity test nevertheless did not show any difference. Furthermore, measurements of the sample weight before and after two months on shelf indicated a 3.16 ± 0.75% mass decrease for the samples without coating, 3.82 ± 0.45% mass decrease for the samples for the samples with the spray and 1.29% mass decrease only for the sample coated with beeswax. Although these results would suggest that the beeswax retain the most water and should therefore be weaker, the mechanical properties show the opposite trend. A reason for this could be the drying of the thin layer of the coating which might create a stiffer envelop to the composite. The samples with the waterproof spray coating showed the largest variability, maybe due to an inhomogeneous application of coating coming from the spraying procedure.

Furthermore, the elastic modulus of samples coated with beeswax increased from 60 to 80 kPa and from 40 to 60 kPa for the samples coated with the waterproof spray (Fig. 4d). In turn, the modulus of the samples without coating slightly decreased from 48 to 40 kPa. This variation in modulus explains the increase in stresses in the low strain region of the stress–strain curves. It is likely that the increase in the stiffness of the composites led to the disappearance of the visible yield which was a shoulder in the curve.

Overall, we found that the MBCs were completely durable during these 2 months and that the coatings even improved their stiffness. Other coatings could be applied for coloring or other purposes, depending on the intended application. The absence of visible decay of the material and the stability of the mechanical properties suggest that the durability of MBCs indoors can be extended further. Indeed, coated samples can be kept for longer times indoors, provided they are handled carefully, as reported by other works^49,50^. Also, we found that the mechanical properties of the dried samples were lower than that during the growth (phase 2 of the cycle). An explanation for the lower properties could be a variation between batches of samples. As a conclusion, the time for ${t}_{3}$ for the phase 3 is considered to be at least 2 months.

#### Phase 4: biodegradation in soil

**Figure 5:**
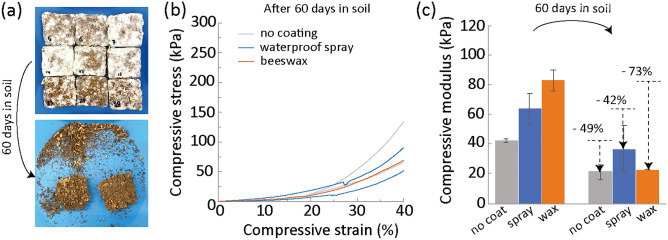
Composting of MBCs with no coating (grey), waterproof spray coating (blue) and beeswax coating (orange). (**a**) Pictures of the MBCs before and after 2 months in soil. Width of the samples is 3 cm for all. All samples looked identical irrespective of their coating. (**b**) Stress–strain curves obtained under compression for the mycelium-bound composites after 2 months in soil. (**c**) Elastic modulus of the samples before and after 2 months in soil.

After using the MBCs, we envision them to be discarded as compost in soil. Indeed, composting is a common approach for managing waste. Also, composting enables the biocycle to join other biocycles as spent mushroom substrates have been reported to serve as fertilizer, soil amendment, biofuel, bioremediation, etc^51^. To demonstrate phase 4 of the biocycle, coated and uncoated MBCs were placed into a porous teabag pouch into soil and submitted to outdoors tropical weather conditions (Fig. 5).

After leaving them for two months in soil, the pouches were dug out and opened. All samples showed biodegradation (Fig. 5a). The white fungal skin covering the sample and binding the substrate components together was not visible anymore on any sample. As a consequence, a number of samples could not hold together anymore and were powdery. Those samples could not be tested in compression. Partially degraded bamboo microfibers from the substrate remained. The biodegradation of the mycelium was likely conducted by the proliferation of bacteria and other organisms feeding on proteins, chitin and cellulose, the main components of mycelium^52^. The bamboo fibers were still present after two months likely because they still contained a high amount of lignin which takes longer to decay in soil^53^. The samples without coating had a 35.89 ± 3% mass decrease, the samples with spray coating had a 44.00 ± 2.6% mass decrease and the sample with beeswax, a 64.13 ± 1.9% mass decrease.

The remaining samples that held together were tested under compression (Fig. 5b). Again, the compression curves exhibited a densification behavior. The stresses however were significantly lower than for the fresh MBCs, as expected from the degradation. No yield point was visible in the curves, likely due to the absence of the mycelium network.

The elastic modulus after degradation were significantly decreased as expected (Fig. 5c). After degradation, the modulus of the non-coated samples decreased by 49%, 42% in the samples coated with the waterproof spray, and 73% in the samples coated with beeswax. Beeswax being a natural material composed of monocarboxylic acids, fatty acids, and hydrocarbons^54^, it has been reported to stimulate the biodegradability of petroleum oils and woods^55–57^. However, only one sample could be tested for the beeswax coating as the others became too powdery for a compression test. Therefore, the difference to the other samples may not be significant. After degradation, the elastic modulus of the specimens was about the same, of 25 kPa in average.

These results indicate that it takes less than 2 months to degrade mycelium by composting MBCs in soil in tropical climate. The degradation was likely conducted by microbial activity and can be expected to be accelerated in presence of more water and physical crumbling of the substrate prior to its composting. In colder climates, the degradation might be slower, especially during winter. The time ${t}_{4}$ for this phase of the biocycle is therefore less than 2 months. After its decomposition, the mycelium releases nutrients into the soil, including lignocellulosic enzymes, hydrocarbons and nitrates^23,24^, which can serve as fertilizers for the phase 1 of the biocycle.

### Second and subsequent cycles

To start a new biocycle, one way could be to totally degrade the previous MBC and start with a fresh substrate. Another strategy could be to take a MBC that has been already used and broken, and to use it as a substrate to regrow a new MBC^9,25^. Here, we investigated an intermediate scenario where an MBC that was kept indoors for 2 months and undergone compression test was crumbled and mixed with freshly made substrate. Doing so allows the integration of this biocycle with other biocycles to extend the total duration. For example, the bamboo grown could be used as poles for construction or other bamboo-based products, while waste generated by cutting the poles to the right dimensions is used to produce MBCs. Alternatively, the bamboo poles could be used in scaffoldings and other applications and converted into bamboo microfibers for mycelium substrates only at the end of their lifetime. Mixing fresh and new substrates with older samples avoids the complete depletion in nutrients that would inevitably occur if the same substrate were recycled over a large number of cycles.

**Figure 6:**
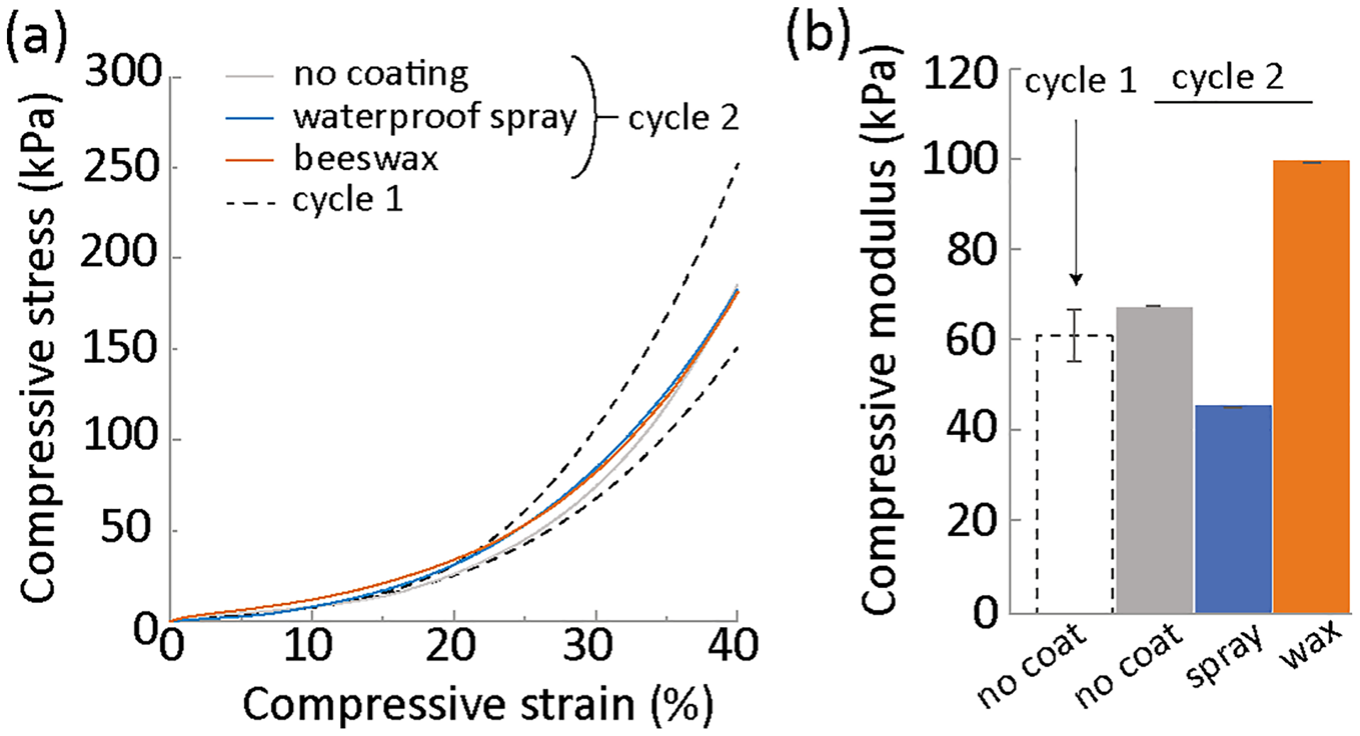
Compression tests of MBCs made from 50% substrate recovered from a used, compressed composite that had no coating (grey), the waterproof spray coating (blue) and the beeswax coating (orange) and compared to the fresh MBCs (dotted line). (**a**) Stress–strain curves obtained under compression after 28 days mycelium growth. (**b**) Elastic moduli.

We therefore produced new MBCs by combining the bamboo microfibers recovered after the compression tests indoors with fresh microfibers at a ratio 50 to 50. After growing new composites for 28 days, compression tests were again conducted without applying any coating (Fig. 6).

Mycelium could grow as expected on the mixture of recycled and new substrate. The samples were grown for 28 days before being tested after drying and without applying any coating. The stress–strain curves obtained for the composites grown from the bamboo fibers recovered from the compressed non-coated mycelium composites and those with the waterproof spray and beeswax coatings were undistinguishable (Fig. 6a). The curves were also similar to those obtained in the first cycle.

The modulus of the samples in the second cycle were not significantly different from those of the composites in first cycle although an effect of the coating is visible (Fig. 6b). The modulus of the samples coated with the waterproof spray was slightly lower which might be due to the presence of toxic chemical compounds. In turn, the samples made from the recycled substrate from composites with a wax coating showed significantly higher modulus, reaching to 100 kPa. However, due to the limited number of samples tested, this difference may not be significant.

These results show that mixing fresh and used substrates can be used for the MBC to enter a new cycle.

### Mycelium-bound composites biocycles

**Figure 7:**
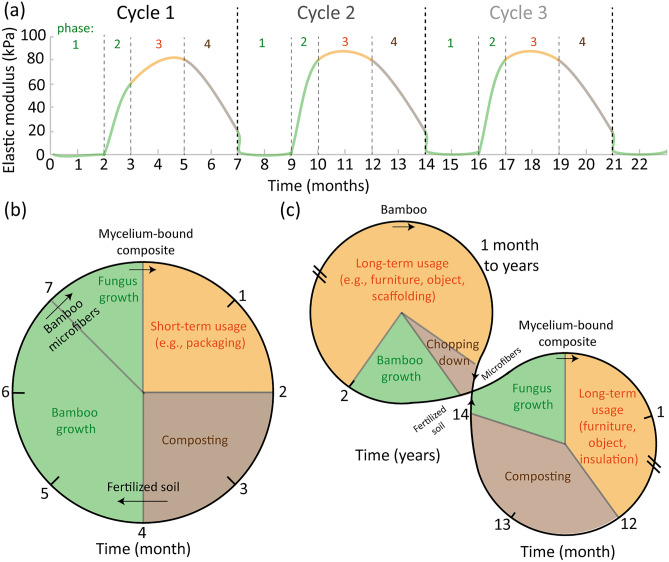
MBC biocycles. (**a**) Elastic modulus as a function of time for 3 consecutive cycles. The lines are guides to the eye. (**b**) Biocycle clock based on the experiment carried out in this study, which is suitable for mycelium composites used for packaging, for example. (**c**) Hypothetical biocycle for MBCs used in a durable application and integrated with another biocycle from bamboo.

We have measured experimentally tangible times for each phase of a MBCs biocycle by measuring the variation in modulus at each phase of the cycle (Fig. 7).

Overall, we found that one biocycle could be completed in about 7 months (Fig. 7a). The biocycle clock contains 4 phases (Fig. 7b). The first phase has an approximative time of 2–3 months and is the growth of the bamboo grass to obtain the microfibers to use as substrate for the fungus growth. This time ${t}_{1}$ could be reduced to zero by integrating with other products related to bamboo industry, such as for scaffolding, food, and others, and using the waste materials as the substrate (Fig. 7c). The second phase of the biocycle is the growth of the fungus to produce the MBC. This phase lasts between 0.5 and 1 month. Although the growth time can be shortened by addition of supplements and optimization of the growth conditions, 1 month is the maximum growth time reported for making such composites. The third phase is when the composite is used in its application. Although the mechanical properties of our MBCs in this study are very modest, they may still be used as packaging, for example. In the case of stronger composites, such as those obtained after hot pressing, the biocycle can be expected to be similar although a careful degradation study should be conducted. Furthermore, for an application requiring durability such as for the use of MBCs as decorative objects, pieces of furniture, or sound-absorbing indoor panels, the usage time ${t}_{3}$ would ideally be as long as possible (Fig. 7c). Appropriate coatings and pressing of the composites could lead to a lifetime indoors of several years. The last phase of the cycle is the biodegradation which could also be accelerated, for example by submerging the composites in water under agitation or by making use of a composter.

## Conclusions

In summary, we conducted proof-of-concept experiments to demonstrate mycelium-bound composites biocycles that include the growth of the bamboo plant for making the bamboo microfibers used as the substrate, the growth of the fungus, the usage of the MBC and its composting. We found that such simple biocycle can be as short as 7–8 months for a short-term usage of MBCs for applications like packaging. In turn, the biocycle could be extended to much longer time for long-term indoor application as an object, a piece of furniture or an insulating panel, for example, after using a hydrophobic protective coating. By testing a waterproof commercial spray and a natural beeswax coating, the latter one appeared to stimulate the degradation while increasing the mechanical properties during shelf-life indoors. Finally, we further suggest that the life cycle of MBCs can be also made more complicated into a double-loop life cycle where the bamboo microfibers are a side product from another biocycle, like the one of bamboo processing for scaffoldings, objects, etc. Better understanding the duration of MBCs biocycles will enable us to envision a viable sustainable economy where individual biocycles connect to each other, like here the MBC biocycle and the bamboo biocycle. Connecting biocycles together will permit to create a biocycle with multiple ‘loops’ where the end life of a product from one loop becomes the fuel of the next loop.

The experiments were conducted under tropical climate in Singapore, and it would be interesting to conduct similar work under other climates. The results from this study could also be complemented by a more complete chemical analysis of the biodegraded materials, especially when various coatings are used in application, and the use of ASTM standard to compare with other biodegradable materials. For example, the MBCs could be used as filters to remove toxic metallic ions from water. In these cases, the MBCs used may not be appropriate for composting as they may contaminate the soil. Similarly, compressed MBCs may degrade with a different rate. Overall, MBCs are exciting materials to pursue sustainable bio-economies and reduce the depletion of natural resources in a variety of applications.

## Data Availability

The data that support the findings of this study are available from the corresponding author, HLF, upon reasonable request.
